# Properties of the Extracellular Polymeric Substance Layer from Minimally Grown Planktonic Cells of *Listeria monocytogenes*

**DOI:** 10.3390/biom11020331

**Published:** 2021-02-22

**Authors:** Ogueri Nwaiwu, Lawrence Wong, Mita Lad, Timothy Foster, William MacNaughtan, Catherine Rees

**Affiliations:** 1 Division of Food, Nutrition and Dietetics, School of Biosciences, University of Nottingham, Nottingham LE12 5RD, UK; lawrence@lierda.com (L.W.); mita.lad@nottingham.ac.uk (M.L.); tim.foster@nottingham.ac.uk (T.F.); bill.macnaughtan1@nottingham.ac.uk (W.M.); cath.rees@nottingham.ac.uk (C.R.); 2 Senthink Science & Technology, Hangzhou 310000, China

**Keywords:** extracellular polymeric substance, capsule, *L. monocytogenes*, minimal media, proteinaceous and non-proteinaceous moiety, spectroscopy, bond stretching

## Abstract

The bacterium *Listeria monocytogenes* is a serious concern to food processing facilities because of its persistence. When liquid cultures of *L. monocytogenes* were prepared in defined media, it was noted that planktonic cells rapidly dropped out of suspension. Zeta potential and hydrophobicity assays found that the cells were more negatively charged (−22, −18, −10 mV in defined media D10, MCDB 202 and brain heart infusion [BHI] media, respectively) and were also more hydrophobic. A SEM analysis detected a capsular-like structure on the surface of cells grown in D10 media. A crude extract of the extracellular polymeric substance (EPS) was found to contain cell-associated proteins. The proteins were removed with pronase treatment. The remaining non-proteinaceous component was not stained by Coomassie blue dye and a further chemical analysis of the EPS did not detect significant amounts of sugars, DNA, polyglutamic acid or any other specific amino acid. When the purified EPS was subjected to attenuated total reflectance-Fourier transform infrared (ATR-FTIR) spectroscopy, the spectra obtained did not match the profile of any of the 12 reference compounds used. An x-ray diffraction (XRD) analysis showed that the EPS was amorphous and a nuclear magnetic resonance (NMR) analysis detected the presence of glycerol. An elemental energy dispersive x-ray (EDX) analysis showed traces of phosphorous as a major component. In conclusion, it is proposed that the non-proteinaceous component may be phospholipid in nature, possibly derived from the cell wall lipoteichoic acid.

## 1. Introduction

The organism *Listeria monocytogenes* is one of the two pathogenic species among the genus *Listeria* [1] that has 20 species [2]. It is known to cause severe foodborne infections in humans and animals. Although the reported occurrence of listeriosis is rare compared with other foodborne infections, the fatality rate is high [3,4] and the economic losses associated with an outbreak of listeriosis support the need for active surveillance and control measures [5].

The persistent presence of *L. monocytogenes* has been widely reported in food processing plants. Among others, the organism has been found in processing plants of meat [6,7] ready-to-eat fish [8], cheese [9] and vegetables [10]. The strains recovered from the processing environment can be resistant to sanitation chemicals [11] and show multi-drug resistance [12]. To help understand why *L. monocytogenes* may persist in food industry equipment and premises, Carpentier and Cerf [13] suggested a conceptual model of persistence based on the relative weight of growth and the outcome of cleaning and disinfection, which requires a minimum initial bacterial load necessary for bacteria to persist in harbourage sites. It is believed that after sanitation procedures, cells that are not recovered by standard culture methods may remain in the environment [14]. Persistent strains that are believed to be better adapted to stress conditions can form biofilms and harbourage sites caused by difficult to clean equipment, which can also facilitate persistence [15].

The extracellular polymeric substance (EPS) is produced by microbes as secretions of biofilms, which can secure attachment [16]. It has been pointed out [17] that the EPS can form a complex network that aids the enclosed adhered cells to develop three-dimensional structures consisting of both viable and non-viable cells as well as the secreted polymeric substances. The EPS is normally associated with the formation of biofilms when micro-organisms are embedded in the three-dimensional, gel-like hydrated matrix [18]. The EPS is believed to facilitate the survival of micro-organisms with their modes of organisation in different physicochemical states [19]. Hence, the EPS is responsible for the structural and functional integrity of biofilms and is also considered to be a key compound that determines the physicochemical and biological properties of biofilms [20]. When embedded in a biofilm matrix, the EPS is known to have a physical role in protecting the cells against dehydration. It is also believed that the adhering properties of the EPS are important in cell aggregation and floc formation and enable the cells to attach irreversibly to a surface [21]. *Listeria* spp. are not considered to be capsulated bacteria but have been reported to form biofilms and, unlike the situation for many other bacteria, the nature of the EPS material has not been established. As previously reviewed [22], the composition of the *Listeria* EPS has been proposed to consist of a range of different materials including polysaccharides, proteins and extracellular DNA.

The link between the EPS and biofilms is unarguable but sometimes both are lumped together and may be misconstrued as biofilms. The distinction between the two entities has been elucidated by Grigore-Gurgu et al. [23] and while hundreds of genes associated biofilm regulation were presented in their report, specific genes associated with the EPS were not explicitly shown because few studies on the EPS have been carried out in the literature. The production of the EPS upon bacterial adhesion to surfaces is believed to be a biological process that contributes to the strengthening of the bacterium-substratum bond and is a key element in understanding the biofilm phenotype [24]. Therefore, the current study was undertaken to investigate the changes associated with growing *L. monocytogenes* strains in defined minimal media and to establish the EPS properties that would be of relevance to the persistence of these bacteria in food processing plants.

## 2. Materials and Methods

### 2.1. Bacteria Strains and Media

The *L. monocytogenes* strains were grown in either (BHI) broth (Oxoid, Hampshire, UK) or a D10 defined medium to obtain a final pH of 7.2–7.5 [25]. The defined medium consisted of chemically defined compounds that are used for growth and the exact composition was known. If the defined media was minimal, it contained the minimum amount of nutrients required for bacteria growth and would normally not contain amino acids. Cells were also grown in the tissue culture medium MCDB 202 (US Biologicals, Salem, MA, USA) supplemented with a 1% yeast nitrogen base without amino acids and 3.6 g of glucose and was prepared as described by others [26]. Liquid cultures were incubated at 37 °C with shaking and growth was monitored using optical density (600 nm). Widely available laboratory *L. monocytogenes* strains were used; namely, 10403S (serotype 1/2a), EGD (serotype 1/2a), LO28 (serotype 1/2c) and ATCC 23074 (serotype 4b). In addition, a collection of *L. monocytogenes* environmental strains Lm 00054-0305 (serotype 1/2b; from vegetables), Lm 000622-0305 (serotype 1/2a; environment), Lm 00048-0305 (serotype 1/2c; roast chicken) and Lm 000101-0305 (serotype 4b; pot wash) were used. The molecular serotype of these strains was determined [27] by extracting DNA [28] using the laboratory strains as reference samples.

### 2.2. SEM of Cells Grown in Different Media

SEM was carried out by growing cells for 18 h in MCDB, BHI and D10 broth. The cells were then harvested by spinning at 3000× *g* for 15 min and fixed in acetone (Fisher Scientific, Loughborough, UK) for 1 h. A drop of cell/acetone mixture was placed on the SEM stubs and allowed to dry. A drop of tetramethylsilane (Sigma–Aldrich, Gillingham, UK) was placed on the stub to enhance and complete the drying after which the stubs were coated with platinum by spurting (Quorum Technologies, Polaron SC7640, Lewes, UK) for 90 s. The stubs were finally viewed under a high vacuum with a field emission gun SEM (Philips XL 30, Eindhoven, The Netherlands) capable of delivering 10–100,000× magnification and a maximum resolution under optimal conditions of 3.5 nm. SEM evaluation was also carried out on the purified polymer material and an elemental analysis was performed on the different regions of a stub by switching to the energy dispersive x-ray elemental (EDX) trace mode of the machine.

### 2.3. Hydrophobicity, Zeta Potential and Attachment Assays

Cells of *L. monocytogenes* were inoculated into BHI, D10 or MCDB 202 media and grown with shaking at 37 °C for 18 h. A standard microbial attachment to hydrocarbon (MATH) assay with an NaCl buffer as described by other workers [29] was carried out by using 50–500 µL volumes of n-octane, hexadecane and chloroform. Using the same reference strains mentioned above, the tests were carried out on four strains obtained from molecular serotyping. The charge on cells of *L. monocytogenes* when grown in different conditions was measured by growing cells in the three different media used above with shaking at 37 °C for 18 h. A sample (5 µL) of the cells grown in each medium was placed on the reader of the zeta potential instrument (Beckman Coulter Version 1.34, Indianapolis, IN, USA) and readings from three separately prepared cultures were recorded. The cell attachment was measured using the crystal violet (CV) methods of previous investigators [30]. The ratio of attachment relative to the optical density (OD 600 nm) of the input culture was determined by dividing CV OD/OD 600 nm. The interaction of strains, media, solvent type and volume was modelled using Design Expert™ ver. 7.0.3 software, Statease, Minneapolis, MN, USA.

### 2.4. Extraction and Purification of Capsular Polymer from L. monocytogenes

An EPS extraction from *L. monocytogenes* strain Lm 10403S was achieved using the method for poly-gamma-glutamic acid (PGA) extraction [31]. Cells were grown overnight in D10 media and then centrifuged (15,000× *g*, 4 °C, 10 min). Four volumes of absolute ethanol (−20 °C) were added and the EPS expressed in the supernatant was precipitated for 18 h at −20 °C. Pellets were dissolved in 1/60 volume sterile distilled water (SDW) and lyophilised (Telstar, Dewsbury, UK) under a vacuum for 18 h at −85 °C. The dry powder was re-suspended in 1/3000 volume of SDW and then centrifuged (15 min, 20,000× *g*, 4 °C) to remove the insoluble material. The supernatant was dialysed (Float-A-Lyzer^®^, SpectrumLabs, New Brunswick, NJ, USA.) for 18 h against SDW and lyophilised again before the purified EPS powder was reconstituted at 5 mg mL^−1^ in SDW. After pronase treatment (2 µg mL^−1^, 18 h, 42 °C) the enzyme was removed using a 100,000 kDa MW cut-off filter (Microcon™; Merck, Dorset, UK). The treated samples were dried by lyophilisation and reconstituted in SDW at 5–30 mg mL^−1^.

### 2.5. Sodium Dodecyl Sulphate Polyacrylamide Gel Electrophoresis Analysis of Listeria Crude Extract (Purified)

The bands of the capsular EPS were viewed by carrying out SDS-PAGE [32]. The reconstituted capsule solution was used or treated with pronase (Boehringer Manheim Welwyn Garden City, UK) before running on a 10% gel. Untreated samples served as the control. The separating and stacking gels were polymerised for 40 min by the addition of ammonium persulphate (Sigma, Gillingham, UK) and N,N,N,N-tetramethyl ethylene-diamine (TEMED, Sigma, Gillingham, UK). The gel was run in a tank (Bio-Rad, Watford, UK) containing running buffer (25 mM Tris, 0.19% 50S, 0.192 M Glycine (pH 8.3–8.6 without adjustment)) at 200 V for 3 h. The gel was then either stained in Coomassie brilliant blue or with 0.5% methylene blue in 3% acetic acid.

### 2.6. Amino Acids and Sugar Analysis of Crude/Purified EPS Extract

An amino acid analysis was performed using an amino acid analyser (Agilent, Santa Clara, CA, USA) with an ion exchange column. Samples (approx. 300 mg) of the EPS of *L. monocytogenes* before pronase treatment or commercial PGA (Vedan Corp., Taichung, Taiwan) were prepared and loaded onto the amino acid analyser as recommended by the manufacturer. A sugar analysis was carried out after acid hydrolysis (1 M H_2_SO_4_, 100 °C) and sugars were detected using a Sucrose/D-glucose/D-fructose kit (Boeringer Mannheim Cat No 716260, Welwyn Garden City, UK). Complex sugars were analysed as in reference [33].

### 2.7. High Resolution Liquid State Nuclear Magnetic Resonance (NMR)

NMR spectra were acquired at 298 K on a Bruker Avance (III) 500 spectrometer equipped with a dual ^1^H/^13^C cryoprobe (Bruker AXS, Coventry, UK). The dry crystals were dissolved in D_2_O to a concentration of 5 mg mL^−1^ for ^1^H NMR or 30 mg mL^−1^ for ^13^C NMR. ^1^H NMR spectra were acquired with the pre-saturation of the solvent signal using a 2 s relaxation delay and a 90-degree pulse. ^13^C NMR spectra were acquired with a relaxation delay of 1.5 s using 30-degree pulses.

### 2.8. X-ray Diffraction Evaluation of the EPS of L. monocytogenes

X-ray diffractograms of the EPS of *L. monocytogenes* were recorded on powdered samples for values of 2 θ between angles 4° and 38° at 0.1° intervals with a scanning time of 6 s per interval using a Bruker D5005 (Bruker AXS, Coventry, UK) diffractometer at 20 °C. A phase analysis and the identification of crystalline forms were achieved using Bruker Diffrac^plus^ and Eva software coupled to x-ray identification databases.

### 2.9. Attenuated Total Reflectance-Fourier Transform Infrared (ATR-FTIR) Spectra of the Purified EPS of L. monocytogenes

ATR-FTIR spectra of the purified EPS of *L. monocytogenes* were recorded using a Bruker spectrometer (Tensor 27, Bruker AXS, Coventry, UK), with a heated single reflectance diamond Golden Gate^TM^ (Graseby Specac, Kent, UK). After the preliminary test of the EPS, the profiles of bovine serum albumin (BSA), PGA, pectin and DNA from the strain from which the EPS was obtained were compared [34] and a larger batch of compounds was analysed. The new batch consisted of DNA from strain Lm 10403S, BSA, glycerol and components from the defined media, which included aluminium chloride, sodium hydroxide, sodium dihydrogen phosphate, ferric chloride and potassium phosphate. Some of these compounds were run as a check for impurities. Additional comparisons were made with sugars; namely, galactose, sucrose, mannose and glucose.

## 3. Results

### 3.1. Initial Detection of the EPS in Planktonic Cells

**Figure 1:**
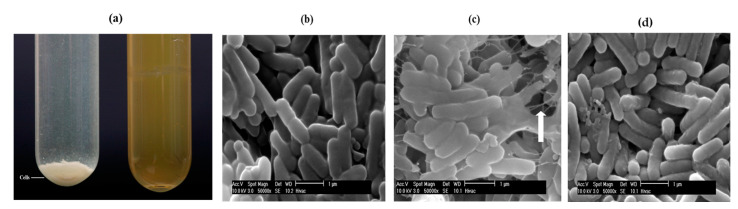
Cells of *L. monocytogenes* (Lm1040S) grown in MCDB 202 (showing flocculated cells) and brain heart infusion (BHI) media at 37 °C for 18 h (**a**). SEM micrographs were captured for cells grown in BHI (**b**) and D10 (**c**) and MCDB 202 (**d**) media. Cells were grown for 18 h with shaking at 37 °C and then harvested from the broth by centrifugation. Cells were re-suspended in PBS, fixed on electron stubs and coated with platinum before viewing. The white arrow (**c**) indicates an area where a thick layer of an extracellular polymeric substance (EPS) can be seen joining cells.

*L. monocytogenes* were grown in three media, BHI (nutrient rich) and two defined minimal media; namely, D10 and MCDB 202. After an overnight incubation, there were clear differences in the distribution of the cells in the different broths (Figure 1a). In BHI media the cells primarily remained in suspension whereas in minimal media the cells tended to drop out of suspension and formed pellets that were easily dispersed by vortexing. This flocculation phenotype was more pronounced in MCDB 202 minimal media (Figure 1a). Samples from liquid cultures of *L. monocytogenes* were initially stained and imaged under the light microscope and the cells seemed to be surrounded by a capsular material. When the samples were also imaged by SEM, cells grown in the BHI broth appeared as single rods with little evidence of EPS formation (Figure 1b). However, cells grown in both minimal media had a visible coating of an EPS with strings of the EPS seen connecting the cells in D10 (Figure 1c) and a more uneven coating of the material on the surfaces of cells grown in MCDB 202 (Figure 1d). The production of this capsular material was strongly associated with growth in the more nutrient limiting media provided by the defined growth media (Figure 1c).

### 3.2. Cell Charge, Hydrophobicity and Attachment of Cells

The ability of bacteria to attach on surfaces is influenced by the cell surface charge and its hydrophobicity [35]. To determine if the production of this EPS material by planktonic cells was likely to affect these cell characteristics, cell charge, hydrophobicity and attachment were measured. Well-characterised laboratory strains of *L. monocytogenes* were chosen but, to extend the study and to address the issue of strain variation, strains of *L. monocytogenes* that had been recently obtained from a commercial bakery of a known molecular serotype were also included.

**Figure 2:**
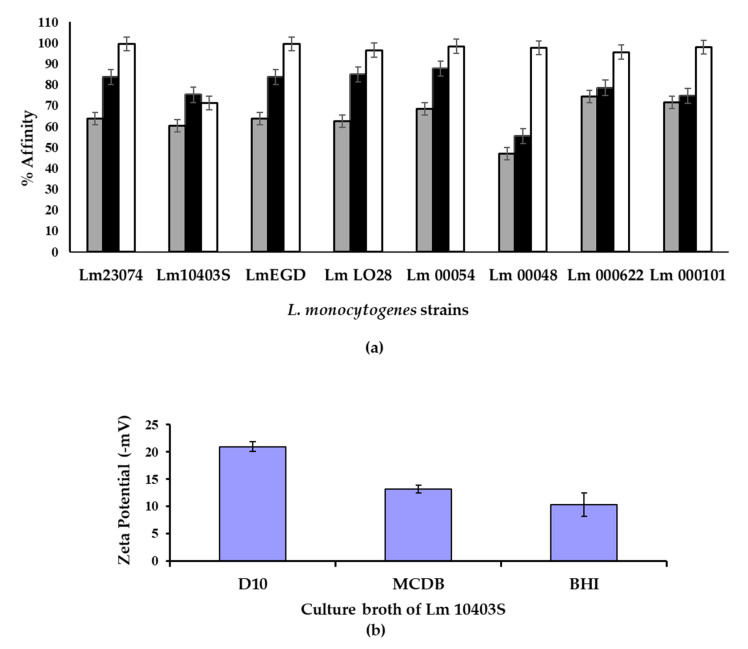
Representative cell hydrophobicity (**a**) assay showing affinity to chloroform solvent (500 µL) and zeta potential measurements (**b**) of strain Lm 10403S cells. Cells (**a**) were grown in MCDB 202 (□), D10 (■) and BHI (■) media before an affinity analysis was carried out with the microbial attachment to hydrocarbon (MATH) method. The zeta potential (**b**) was measured and the results represent values from triplicate independent samples. Error bars represent two standard errors in both panels. Statistical modelling showed that the solvent type and volume, media and strain interaction were significant terms (*p* < 0.05).

Hydrophobicity was determined using the MATH assay by monitoring the partitioning of cells in hydrocarbon as previously described [29]. In all cases, cells that were grown in minimal media (MCDB 202) had the highest affinity for the solvent used. This pattern was seen for all serotypes and both laboratory and recent environmental isolates (Figure 2a). In addition, the surface charge of the cells was determined using zeta potential measurements. Cells grown in the defined D10 media were found to be more negatively charged than cells grown in MCDB 202 or BHI media (Figure 2b).

The attachment of cells to a surface was assessed using a crystal violet microtitre plate assay [30]. As the different strains exhibited different levels of growth in the different media, the level of attachment was normalised by comparison with the concentration of cells present in the inoculum. When the attachment per cell mass of inoculum was recorded there was no evidence that the cell number alone caused increased levels of cell attachment. Overall, cells grown in D10 produced higher levels of attachment per cell concentration (1.21 ± 1.74 CV/OD600 nm) than cells grown in BHI (0.75 ± 0.52 CV/OD600 nm) or MCDB 202 (0.44 ± 0.13 CV/OD600 nm) and this pattern was seen for both laboratory cultures and recent environmental isolates. Considering the fact that cells grown in D10 media had the highest charge and cells grown in MCDB 202 had the highest hydrophobicity, it was concluded that the surface charge may have more impact than surface hydrophobicity in promoting the attachment of *L. monocytogenes* to surfaces. Finally, the statistical modelling performed found that that solvent type and volume, media and strains were significant model terms (*p* < 0.05).

### 3.3. Initial Comparison of the EPS of L. monocytogenes with PGA

**Figure 3:**
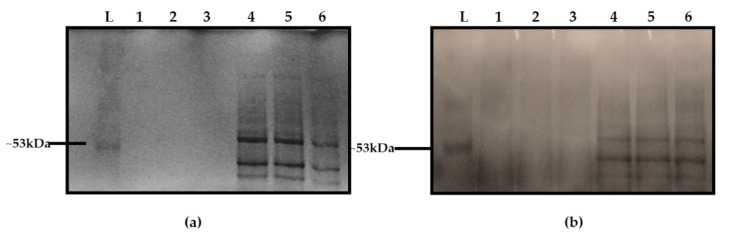
Staining of the EPS of the *L. monocytogenes* crude extract with Coomassie blue (**a**) and methylene blue (**b**). Lane L in both panels is the ladder. In panels (**a**,**b**) samples were treated with pronase, (lanes 1, 2, 3) whereas samples in lanes 4, 5 and 6 were not.

To further characterise the EPS observed in the defined media, a method described for the recovery of an EPS composed of poly-gamma-glutamic acid (PGA) from *Bacillus* culture supernatants [8] was used to recover the EPS released from the surface of *L. monocytogenes* cultures grown in D10. The strain 10403S was chosen for EPS extraction because it has been sequenced and the genome is publicly available. Purified EPS crystals treated and untreated with pronase were initially characterised with SDS-PAGE. When stained with Coomassie blue, samples treated with pronase were not detected (Figure 3a, lanes 1, 2, 3) indicating resistance whereas those that were untreated with pronase were stained (Figure 3a, lanes 4, 5, 6). When methylene blue was used, both treated and untreated samples were sensitive and the protein bands on the untreated samples were visible (Figure 3b). No protein band was found in the EPS samples treated with pronase (Figure 3b, lanes 1, 2, 3), which confirmed that cell-associated proteins were digested. The sensitivity of the EPS to methylene blue and resistance to Coomassie blue stain as with PGA was consistent with the hypothesis that the EPS material from *L. monocytogenes* was similar to that of *Bacillus.*

### 3.4. Amino Acid and Sugar Analysis

The EPS of *L. monocytogenes* was then subjected to amino acid and sugar analyses and the results (Table 1a) showed that it contained only trace amounts of glutamic acid, the dominant component for PGA, whereas a commercial sample of PGA contained high levels of glutamic acid as expected, ruling out PGA as the major constituent of the EPS of *L. monocytogenes*. Simple sugars were detected in the EPS of *L. monocytogenes* at low levels with complex sugars being present in only trace amounts (Table 1b).

### 3.5. Microstructure of the Purified EPS

**Figure 4:**
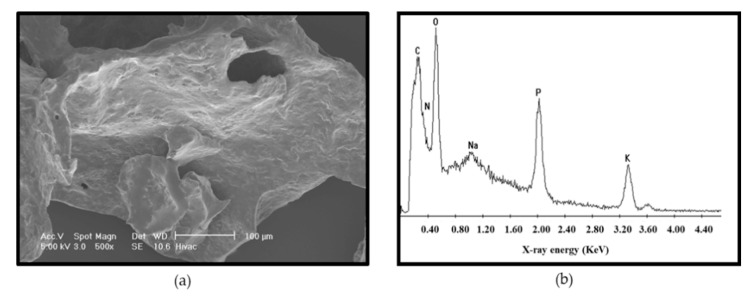
A SEM analysis (500×) of the purified EPS (**a**). The cell culture was pelleted by centrifugation after which the EPS was precipitated, dialysed and treated with pronase to remove cell-associated proteins. The material was freeze dried and stored at −85 °C. Panel (**b**) shows the trace of energy dispersive x-ray elemental analyses of the purified EPS of *L. monocytogenes*.

SEM was carried out on purified extracts from planktonic cells of *L. monocytogenes*. Images of the purified material were free from *L. monocytogenes* whole-cell contaminants and the structure appeared flaky (Figure 4a). The elemental analysis carried out showed carbon, oxygen, sodium, phosphorous and potassium as the dominant elements (Figure 4b).

The most abundant were carbon, oxygen and phosphorous with no nitrogen peaks observed. However, the carbon peak reduced dramatically when areas free from the material on the stub were subjected to elemental analyses. This confirmed the presence of carbon. The lack of nitrogen was significant because it eliminated DNA as the dominant material as the bases in DNA contain significant amounts of nitrogen. Hence, a result of no or low levels of nitrogen indicated that DNA was not the dominant material.

### 3.6. X-ray Diffraction Analysis of the EPS of L. monocytogenes

**Figure 5:**
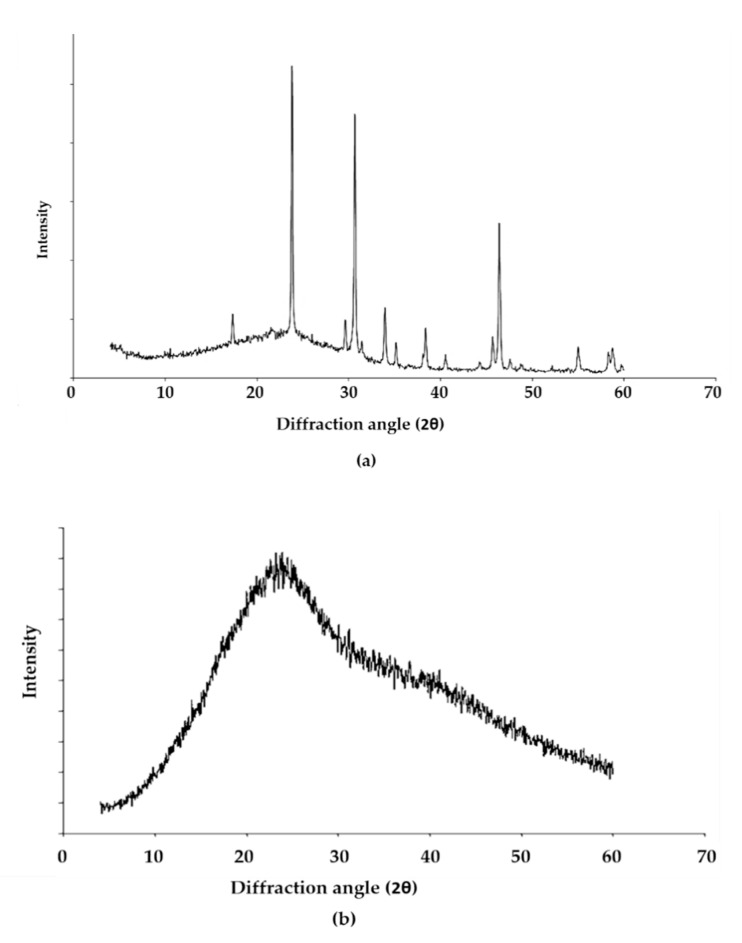
X-ray diffraction traces of PGA (**a**) and the EPS from *L. monocytogenes* (**b**) Diffractograms were recorded on powdered samples with a scanning time of 6 s per angular interval at 20 °C.

In an attempt to gain more structural information, x-ray diffraction was performed on the EPS of *L. monocytogenes* with PGA being used as a comparative crystalline material. The results (Figure 5) showed that the EPS was amorphous with no discernible crystalline components in contrast to the crystalline PGA. The broad featureless hump between 12 and 36 degrees indicated the presence of an essentially amorphous material.

X-ray diffraction when conducted on crystalline phases is a powerful method of compound identification. However, in the absence of crystalline fractions, the method loses all power and it becomes impossible to say which compounds are present in the EPS as most amorphous fractions look similar. There will, however, still be structure even in apparently structureless materials. For example, if the molecules approximate a random collection of spheres then there will be loose associations of shells of molecules surrounding a central molecule. This was the basis of the radial distribution function analysis. The value of the spacings corresponding to the peaks of the humps could give information on the most likely distances between molecules. However, it was impossible to identify individual compounds and the information clearly was limited to the point of being valueless in this case. What can be said is that there was clearly no crystalline PGA or any other crystalline phases present.

### 3.7. NMR Analysis of the EPS of L. monocytogenes

**Figure 6:**
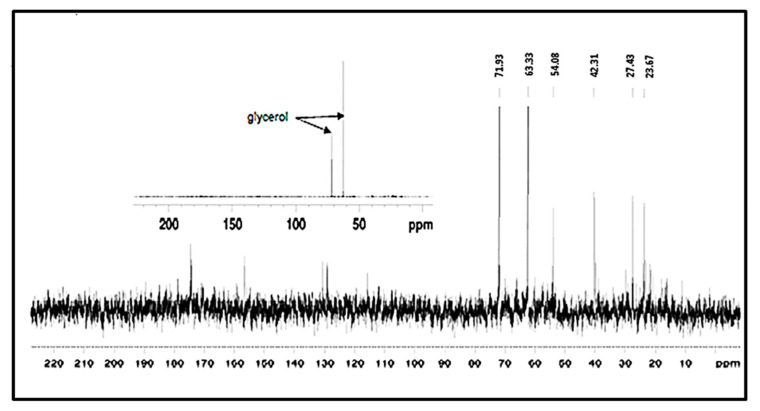
^13^C NMR spectrum of the EPS of *L. monocytogenes*. The EPS of *L. monocytogenes* (30 mg) was dissolved in 4% deuterated water. Spectra were acquired with ^13^C NMR and peaks due to the carbons in glycerol were visible.

A ^13^C NMR spectrum of the EPS of *L. monocytogenes* with approximately 5 mg of sample gave no clear result due to poor signal to noise ratio. However, when the sample size was increased to 30 mg, a signature typical of glycerol (Figure 6) was detected. The identification of this was confirmed by the addition of pure glycerol to the sample, which resulted in an increase in the peak height of the unknown compound, i.e., peaks from glycerol were present in the same position as the EPS. The glycerol could represent the hydrolysis product of a lipid. What was noticed was that the signal was extremely weak and there were other peaks present at low levels. There appeared to be carbonyl peaks present in the 170–180 ppm region (main peak ~175 ppm) and the presence of aliphatic carbons in the region of 15–40 ppm consistent with the presence of fatty acids or residual lipids, once again suggesting components of a lipid origin. The weak signal was felt to be due to the high concentration of non-carbon containing compounds in the EPS, possibly buffer salts arising from the freeze drying of an incompletely dialysed sample, which led to the poor signal to noise ratio and inconclusive nature of these results.

### 3.8. ATR-FTIR Analysis of Purified L. monocytogenes

**Figure 7:**
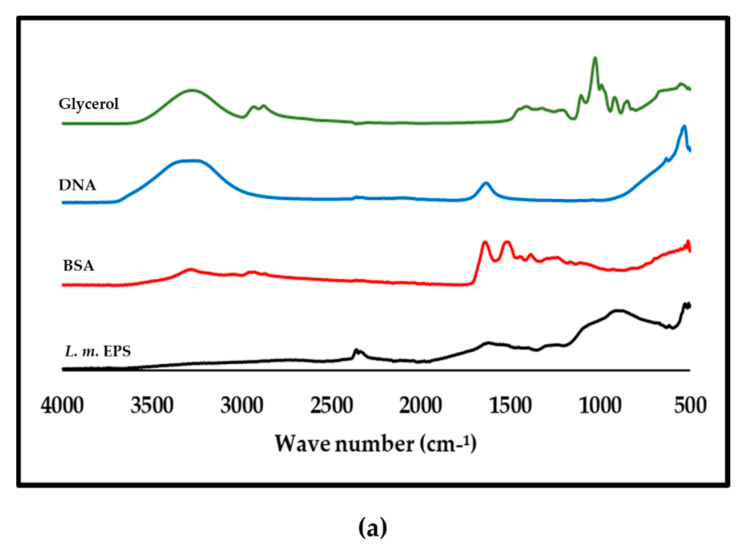

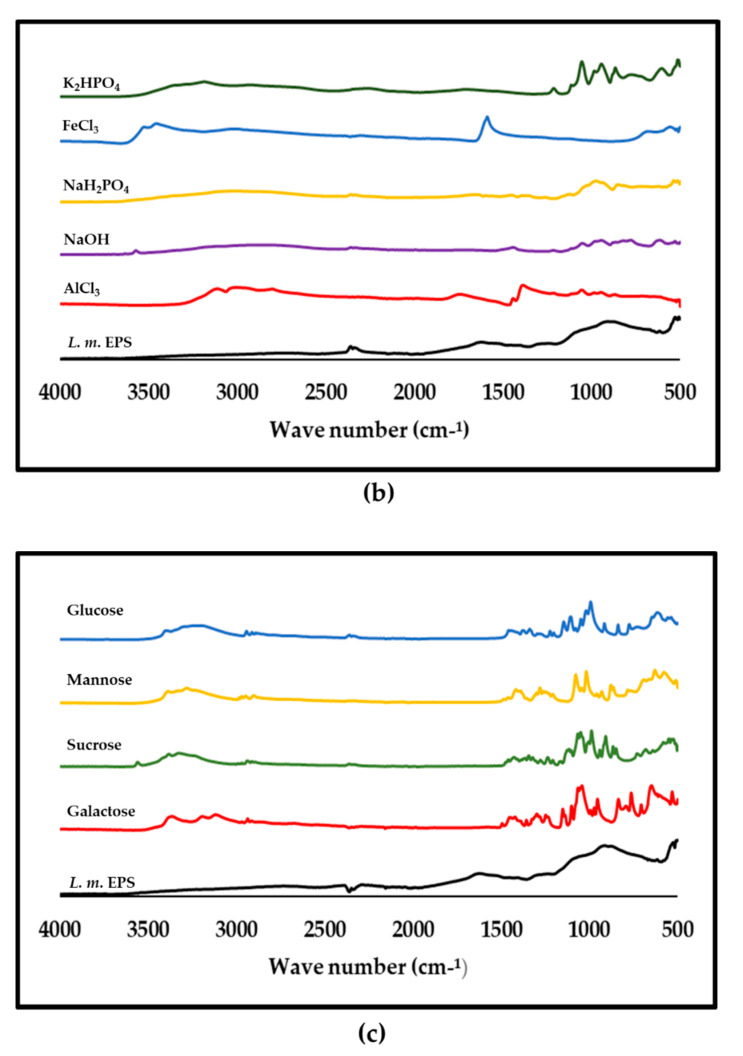
Attenuated total reflectance-Fourier transform infrared (ATR-FTIR) spectra of purified *L. monocytogenes* and other compounds. In panel (**a**), the EPS (the EPS of *L. monocytogenes*) was compared with bovine serum albumin (BSA), DNA and glycerol. Panel (**b**) shows a comparison with the defined media components, which included aluminium chloride (AlCl_3_), sodium hydroxide (NaOH) sodium dihydrogen phosphate (NaH_2_PO_4_), ferric chloride (FeCL_3_) and potassium phosphate (K_2_HPO_4_). These ionic materials were used to test for the presence of impurities. Further comparisons were made with sugars (**c**); namely, galactose, sucrose, mannose and glucose.

From the preliminary FTIR results [34] there appeared to be similarities between the EPS of *L. monocytogenes* and BSA. However, a rerun of the full spectrum (Figure 7a) showed that the profiles were not the same. The double lipid peaks due to C-H stretching in CH_3_ and CH_2_ at approximately 2925 and 2855 cm^−1^ [36] were seen in glycerol but not in the EPS polymer (Figure 7a). No minimal media component tested matched the EPS of *L. monocytogenes* (Figure 7b). The spectra in Figure 7c compared galactose and other sugars with the EPS profile and again no similarities in profile were seen.

## 4. Discussion

In food processing environments, the growth of *L. monocytogenes* on sanitised equipment occurs on a minimal nutrient surface. To monitor the EPS formation under low nutrient conditions, we used two minimal media to grow the organism. One was prepared in the laboratory (D10 medium) and another one (MCDB 202) was sourced commercially. To contrast, cells were also grown in the rich BHI medium.

### 4.1. Cell Hydrophobicity and Surface Charge

Cell hydrophobicity has often been reported as a feature that can influence attachment to surfaces and facilitate persistence in the factory environment. The use of nutrient limited media to grow *L. monocytogenes* resulted in changes that affected the surface properties of the cells as evidenced by the change in hydrophobicity determined by the MATH assay. Cells grown in the two minimal media had higher hydrophobicity or attachment to the solvent used and this agreed with other findings [37] that found that *L. monocytogenes* cells grown in minimal media D10 showed a 50-fold higher attachment to stainless steel than those grown in tryptone soy broth (TSB). Previously, it was found [38] that biofilm formation was greater when *L. monocytogenes* was grown in the tissue culture medium MCDB 202 than in a tryptone soy broth yeast extract (TSBYE) medium whereas cultivable bacteria from a biofilm population on Petri dishes were enumerated in greater quantities in TSBYE than in a MCDB 202 medium.

Of the two minimal media used, a higher hydrophobicity index was recorded for MCDB 202 but it had a lower ratio of attachment relative to the density of the input culture than the D10 minimal media after attachment assays. This indicated that cells grown in D10 media may form more biofilm than those from MCDB 2020. This contrasted with the findings of Doijad et al. [39] where it was reported that a higher hydrophobicity resulted in an increased biofilm formation. The differences may be down to the physiological conditions of the cells that were characterised by the altered surface properties of *L. monocytogenes* in various media after a SEM analysis. The use of SEM to probe the EPS and biofilms on surfaces is now the norm [40] and further studies on the growth of *Listeria* in a different environment will bring more insights into the behaviour of the organism on surfaces.

Changes in the cell surface may promote binding to surfaces and the results demonstrated the need to grow *Listeria* cells in an environmentally relevant medium to ensure that results gained could be translated into real-world situations. However nutrient limitation alone cannot be the only trigger for this alteration in cell surface properties as it has been previously reported that *L. monocytogenes* grown in trypticase soy agar was more hydrophobic than cells grown in the more limited medium peptone agar [41]. This suggests that constituents of the media also play a role in triggering changes in the surface properties of the cell. In line with this idea, Frank and Koffi [42] found that growth in TSB supplemented with glucose promoted the adherence of *Listeria* to glass slides and found that the adherent cells became more resistant to sanitisers. They did not investigate whether this was due to the enhanced production of any extracellular material but did note that LTA (Lipoteichoic acid) production could be stimulated by adherent growth and their lipophilic property might prevent the penetration of sanitisers.

The zeta potential results showed that cells were negatively charged when grown in all media but were most negatively charged in the minimal media. A change in surface charge may affect electrostatic interactions between *L. monocytogenes* cells and surfaces. Our results were in line with a previous investigation that established a link between electrostatic charges and microbial adhesion to hydrocarbons using the MATH assay [29]. The investigation found that regardless of the medium used to cultivate *L. monocytogenes* strain Scott A, the cells always had an affinity for electron acceptor solvents rather than non-polar solvents. It has also been found that bacterial cell surfaces exhibit electron-donating properties regardless of incubation temperature and became more hydrophilic as the temperature decreased from 37 °C to 4 °C [43].

### 4.2. Constituents of the EPS of L. monocytogenes

The propensity for *Listeria* cells in liquid cultures to clump together when grown in standard BHI broth has previously been recognised but this phenomenon became more pronounced when cells were grown in a more nutrient limiting media. When cells recovered from these cultures were imaged by SEM, an EPS layer was clearly evident on the surface of these planktonic cells. The ability to produce capsules by an organism aids attachment but, unlike many of the Gram-positive bacteria, *Listeria* is still typically described as non-capsulated [44]. Older studies reported the identification of a capsule layer by TEM on *L. monocytogenes* cells grown in TSA broth supplemented with 5% (*w*/*v*) glucose and 10% (*v*/*v*) rabbit serum [45] and therefore our results supported these early observations that when grown in media other than BHI, *Listeria* does produce a detectable capsular layer. Using SEM, it is generally known that the EPS is cell surface-bound and the linking of cells by extracellular material shown in this study was consistent with images provided by others [46].

The main constituents reported for the *Listeria* EPS in a review [22] included teichoic acids, proteins and extracellular DNA. To ascertain the nature of the EPS material, a range of analyses were performed because there is no known single method of extracting a dominant constituent of the *L. monocytogenes* cell surface. Hence, it was hypothesised that the EPS of *L. monocytogenes* might be similar to PGA synthesised by *Bacillus.* This is because Sallen et al. [47] found that at the intergeneric level, *Listeria* species grouped in a monophyletic cluster and exhibited the highest levels of sequence similarity with *Bacilli* (up to 95%) among other Gram-positive organisms when a comparative analysis of 16S and 23S rRNA sequences of *Listeria* species was carried out. Bergley et al. [48] also found a homologue of the *Bacillus anthracis capA* gene in *L. monocytogenes* LO28. The gene was reported by Makino et al. [49] to be involved in capsule synthesis in *B. anthracis* and mediates the polymerisation of D-glutamic acid via the membrane. Hence, PGA was included in most assays carried out in this study to establish if there were similarities with biomolecules found on the surface of *L. monocytogenes* planktonic cells. However, the EPS amorphous structure from the x-ray diffraction (XRD) analysis and the fact that glutamic acid was not its dominant amino acid provides compelling proof that the EPS from this study was not PGA. The absence of any significant number of amino acids in the EPS polymer also suggests that it was not a polymer made from another amino acid. The sugar analysis showed trace amounts as observed in other investigations [50].

The results were compared with other molecules that have been implicated in the formation of the EPS layer in *L. monocytogenes*. There was no evidence of significant amounts of extracellular DNA [51], complex sugars [52] or PGA found in *Bacillus* [53] being present in the purified EPS material. The crude extract expectedly contained cell-associated proteins as reported by others [54] but when the EPS was digested with pronase, a resistance to the Coomassie stain was observed that indicated that there was a non-protein component present. It is well known that the Coomassie dye binds to proteins through ionic interactions and Van der Waals attractions between the dye and positive protein amine groups. Hence, a resistance to Coomassie dye binding showed that detectable protein was not left after treatment with pronase. A strong signal for phosphorous after the elemental analysis and the detection of glycerol by NMR was consistent with an LTA/lipid-like material being a component of the EPS as reported by Hether and Jackson [55]. Furthermore, fatty acids, associated with glycerol in lipids, have been reported [39] as a component of *L. monocytogenes* cell excretions. The report of Köseoğlu et al. [56] suggested the presence of galactose. Based on carbohydrate composition, linkage and the NMR analysis, the structure of their purified EPS was identified as a β-1,4-linked N-acetylmannosamine chain decorated with terminal α-1,6-linked galactose. However, after the FTIR analysis, the galactose profile and other sugars did not match the EPS profile detected in this study.

Glycerol metabolism and PrfA activity in *L. monocytogenes* have been studied [57] in detail. It was noted that *L. monocytogenes* can efficiently utilise glycerol as a carbon source and that in a defined minimal medium, the growth rate in the presence of glycerol was similar to that in the presence of glucose or cellobiose. In that study, comparative transcriptome analyses of *L. monocytogenes* showed a high-level transcriptional upregulation of the genes known to be involved in glycerol uptake and metabolism (*glpFK* and *glpD*) in the presence of glycerol (compared with that in the presence of glucose and/or cellobiose). It was also found that the levels of expression of the genes encoding a second putative glycerol uptake facilitator (GlpF_2_) and a second putative glycerol kinase (GlpK_2_) were less enhanced under these conditions. It was concluded that GlpK_1_ but not GlpK_2_ was essential for glycerol catabolism in *L. monocytogenes* under extracellular conditions. In *Listeria*, the synthesis of the polyglycerol backbone of LTA is carried out by the enzyme LtaS on the outer surface of the bacterial membrane [58] and, therefore, disruption of this process may lead to the release of polyglycerol from the cell surface.

The spectra obtained for the EPS of *L. monocytogenes* in the amide I band region (approximately 1640 cm^−1^) that was due almost entirely to the C = O stretch vibrations of peptide linkages [59,60] was much smaller than that present in the BSA sample. This signal was taken to be indicative of a protein secondary structure being detected and its absence confirmed that the purified EPS of *L. monocytogenes* was not predominantly composed of protein. Although similarities were noted between the EPS of *L. monocytogenes* and BSA (Figure 7a) in amide region III–V (1200–600 cm^−1^), care should be taken in interpretation because peaks in this region are regarded as having complex vibrations in the peptide bonds and depend on the details of the force field, the nature of side chains and hydrogen bonding [61]. Therefore, it cannot be concluded that a peak in this region indicated a structural similarity at this stage. Importantly, the EPS of *L. monocytogenes* was also found to have a very different spectrum to that of DNA (Figure 7a), which was previously reported to be a major component of the *Listeria* biofilm matrix with a role in both initial attachment and early biofilm formation [51]. This result was consistent with the elemental analysis performed following SEM imaging (Figure 4b), which showed that that carbon and oxygen were the most abundant elements detected and lower amounts of sodium, potassium and phosphorus, but no nitrogen, were detected supporting the conclusion that DNA was not a major component of the purified EPS.

The NMR in conjunction with the FTIR trace analysis appeared to show a proteinaceous signal (characteristic amide I and II bands), albeit of a weak intensity and a spread nature, along with other lower intensity carbohydrate and probably lipid signals. It may be concluded that this particular EPS sample was a relative mixture of various materials. The relatively simple carbon NMR spectrum of the EPS supported this interpretation; however, a closer examination of the carbon spectrum revealed the presence of many more minor signals so the sample was relatively impure but the signal was consistent with a protein or lipid or both. The simple proteinaceous material detected may be polypeptides broken down after a pronase digest of the proteins contained in the crude extract. The lower intensity of the lipid carbonyl peak, which is normally shifted to higher wavenumbers in the FTIR spectrum, was also consistent with the interpretation of the EPS being predominantly protein or lipid-like. It was suspected that in the extract there was a high concentration of ions present (Figure 4b). This may have been introduced in the sample preparation stage—for example, during lyophilisation of an incompletely dialysed sample—or be a characteristic of the EPS. To establish that there was no heavy contamination from reagents, the compounds used for preparing the minimal media D10 were compared with the EPS and FTIR traces obtained showed no similarities especially when the fingerprint region (1500–500 cm^−1^) was compared (Figure 7b).

Of note is the fact that strong peaks due to the presence of glycerol, which was detected by NMR, was not detected by the FTIR analysis. This may be due to many factors. Combrouse et al. [35] noted that, depending on the viability of the bacteria and the analysis method, the quantities of biomolecules detected were different according to the strains and the medium used. This is consistent with the SEM micrographs obtained that showed clear differences in the surface of *L. monocytogenes* grown in different conditions.

### 4.3. Role of the EPS in the Food Processing Environment

*L. monocytogenes* is thought to be present in various environmental reservoirs and exhibits resistance and tolerance to antibiotics and sanitising agents used in several food processing industries [62,63]. The organism is a concern in food safety [64] because it shows large variations in competitive growth in food processing environments [65]. The need for improvement in preventive measures and a conscious selection and use of sanitisers in food-related environments to control *L. monocytogenes* has been highlighted [66]. Currently, the consensus is that when central extracellular components are enzymatically degraded, the biofilm size can be reduced considerably [67]. Hence, there is a need for a greater understanding of the EPS produced in the food processing environment. Literature is awash with work on the biofilm as a whole but more work that will characterise the EPS and its constituents during growth in the environment is required. Knowledge gained can be used to formulate more efficient sanitising agents.

Suggestions on how to stop EPS and biofilm formation include the potential use of oxygen and nitrogen reactive intermediates [68], the application of photodynamic food-grade photosensitisers [69], the use of combined enzyme-benzalkonium chloride treatments [70], surface programming of EPS production [71] and inhibition by bacteriocin producing bacteria [72,73,74]. The increasing trend in the food industry of *Listeria monocytogenes* biofilm formation is attributed to its easy survival on contact surfaces, resistance to disinfectants or antibiotics and growth under the stringent conditions used for food processing and preservation [75]. In that report, it was emphasised that there is a lack of conclusive evidence about the mechanism of biofilm formation. More knowledge of the EPS is needed to help understand the true mechanisms of EPS and biofilm formation.

## 5. Conclusions

The analysis performed in this study showed clearly that the crude extract was composed of a protein and a non-proteinaceous moiety. The cell surface containing the EPS material was altered when the cells were obtained from different growth conditions. After pronase treatment, it was found that the non-proteinaceous component of the EPS produced by planktonic cells of *L. monocytogenes* in nutrient limiting culture media was not composed mainly of PGA, carbohydrates, DNA or other well-characterised typical EPS materials in literature. It was amorphous and not homogeneous and was resistant to staining by Coomassie blue dye. The study adds to the knowledge of the biomolecules from the surface of *L. monocytogenes* planktonic cells. Understanding the behaviour of *L. monocytogenes* in different growth conditions will help reduce the proliferation of the organism. Further studies on the complex attachment between the *L. monocytogenes* cell surface and various food product contact surfaces in the processing environment are required.

## Figures and Tables

**Table 1:** Amino acid content (g kg^−1^) of the *Listeria* EPS without pronase treatment compared with the amino acid content of poly-gamma-glutamic acid (PGA). Commercial PGA (Vedan, Taiwan) was used for comparison.

**(a)**		
**Amino Acid**	***Listeria* EPS**	**PGA**
Cystine	0.88	0.09
Aspartic acid	1.84	0.34
Methionine	0.38	0.00
Threonine	1.10	0.00
Serine	1.10	0.00
**Glutamic acid**	**3.10**	**193.22**
Glycine	1.72	0.07
Alanine	1.77	0.00
Valine	1.07	0.00
Isoleucine	1.09	0.10
Leucine	1.15	0.00
Tyrosine	1.26	0.00
Phenylalanine	1.24	0.04
Lysine	2.06	0.05
Histidine	0.31	0.00
Arginine	3.96	0.00
**(b)**		
**Sugar Content (% *w*/*w*) of *Listeria* EPS and PGA**		
**Sugar**	***Listeria* EPS**	**PGA**
Sucrose	0.05	1.11
Glucose	0.44	0.09
Fructose	0.06	0.13
Complex sugars	nd	nd

Complex sugars were not detected (nd).

## Data Availability

Publicly available datasets were analyzed in this study. Data sets can be found here: http://eprints.nottingham.ac.uk/13341/ and http://dx.doi.org/10.17632/s8gtdmbx4b.1 accessed on 26 December 2020.
